# Social evolution under demographic stochasticity

**DOI:** 10.1371/journal.pcbi.1006739

**Published:** 2019-02-04

**Authors:** David V. McLeod, Troy Day

**Affiliations:** 1 Institute for Integrative Biology, ETH Zürich, Zürich, Switzerland; 2 Department of Mathematics and Statistics, Department of Biology Queen’s University, Kingston, ON, Canada; University of Pennsylvania, UNITED STATES

## Abstract

How social traits such as altruism and spite evolve remains an open question in evolutionary biology. One factor thought to be potentially important is demographic stochasticity. Here we provide a general theoretical analysis of the role of demographic stochasticity in social evolution. We show that the evolutionary impact of stochasticity depends on how the social action alters the recipient’s life cycle. If the action alters the recipient’s death rate, then demographic stochasticity always favours altruism and disfavours spite. On the other hand, if the action alters the recipient’s birth rate, then stochasticity can either favour or disfavour both altruism and spite depending on the ratio of the rate of population turnover to the population size. Finally, we also show that this ratio is critical to determining if demographic stochasticity can reverse the direction of selection upon social traits. Our analysis thus provides a general understanding of the role of demographic stochasticity in social evolution.

## Author summary

Explaining the evolution of social traits such as altruism and spite remains a key outstanding problem in evolutionary biology. Here we develop a simple theory for the effect of demographic stochasticity (random variation in an individual’s birth and death rates) on the evolution of social traits. Our results provide a clear set of predictions: whether a social trait is favoured or disfavoured is determined by how the social action alters the recipient’s life cycle. If the social action alters the recipient’s death rate, then altruism is favoured and spite disfavoured. If instead the social action alters the recipient’s birth rate, then both altruism and spite can be either favoured or disfavoured—the precise outcome depends upon the ratio of the population turnover rate to the population size.

## Introduction

The evolution of social traits remains a very active area of investigation in evolutionary biology [1–4]. Research has predominately focused upon how different mechanisms such as population structure [5–8], kin discrimination [9–11] or greenbeard effects [3, 12, 13] create heterogeneity in interactions among individuals of different types, leading to the evolution of social traits. Recent evolutionary theory, however, has considered whether or not in the absence of interaction heterogeneity, demographic stochasticity alone can promote the evolution of altruism (e.g., [14–16]; see also [17] for when interaction heterogeneity and demographic stochasticity work in combination). These studies concluded that since altruism increases population size, it confers a stochastic advantage that can reverse (weak) selection against altruism. Counterexamples to this prediction have been found however (e.g., see below) leading one to wonder whether unambiguous conclusions can be drawn.

To address this question we develop a general theoretical analysis of the role of demographic stochasticity in social evolution for well-mixed populations. We start with a detailed description of birth and death events at the individual level [18] and then derive a very simple theory that makes a set of clear, general, predictions. Whether a social trait is favoured or disfavoured by demographic stochasticity is determined by how the social action alters the recipient’s life cycle. When the action alters the recipient’s death rate, altruism is stochastically favoured, and spite is stochastically disfavoured. When the action alters the recipient’s birth rate both altruism and spite can be either stochastically favoured or disfavoured, with the outcome depending upon the ratio of the rate of population turnover to the population size. These results provide a general understanding of the role of demographic stochasticity in the evolution of social traits. They also explain previous models and counterexamples, and illustrate how previous results are special cases of a simple general principle.

## Models

Consider a well-mixed population of size Ω*n*(*t*), where Ω is the habitat size and *n*(*t*) is the population density at time *t*. The population consists of two types of individuals: type 1 individuals, who are social actors capable of altering the birth or death rate of other individuals in the population, and type 2 individuals who are not. The social action may occur through direct contact between individuals or by the production and uptake of an external compound (e.g., the release of siderophores or toxins by bacteria [19–21]). Each individual in the population is equally likely to be the recipient of the social action, and the effect of the social action upon the recipient is identical among types. We distinguish between two possible social traits: *altruism*, which we define to be an action that enhances the vital rates of other individuals (e.g., by increasing birth rates or decreasing mortality rates) and *spite*, which we define to be an action that inhibits the vital rates of other individuals (e.g., by decreasing birth rates or increasing mortality rates). These are standard definitions if the social trait comes at a cost to the actor [22]. Thus at demographic equilibrium the population size *n* will increase as the frequency of altruism increases whereas it will decrease as the frequency of spite increases.

Denote the per-capita birth and mortality rates as *b* and *m*, respectively, and let the per-capita cost of the social trait be *ϵc*, where *ϵ* is a parameter controlling the magnitude of the costs (*b*, *m*, and *c* may depend upon population densities and/or the state of the environment). Thus the per-capita growth rate of social actors (type 1 individuals) and non-actors (type 2 individuals) is *b* − *m* − *ϵc* and *b* − *m*, respectively. As a consequence, whenever *ϵ* > 0, non-actors have a selective advantage, and so in the absence of mutations and stochasticity they will ultimately take over the population. If *ϵ* = 0, then the social trait is cost-free and so neither type of individual is selectively favoured. Finally, we suppose that mutation between the two types occurs at a per-capita rate *μ*. We will further assume that in the absence of selection and mutation, there is an asymptotically stable curve of ecological equilibria given by *b* = *m*. This is a curve rather than a fixed point because in the absence of selection and mutation, both types have identical per-capita growth rates.

If selection is weak, mutations rare, and habitat size large, then the system dynamics occur on two timescales: a fast timescale corresponding to demographic processes (birth and death events) and a slow timescale corresponding to evolutionary change in population composition. As our primary interest is the evolution of the population, our focus is on the slow timescale. On this slow timescale, let *p* be the fraction of social actors (type 1 individuals); then we can rewrite total population density as a function of *p*(*t*) alone, that is, *n*(*t*) = *n*(*p*(*t*)) = *n*(*p*) (see S1 Appendix). Then let *b*(*p*), *m*(*p*), and *c*(*p*) be the per-capita birth, death, and costs on the slow timescale. If *ϵ* is small then *T*(*p*) ≡ *b*(*p*) + *m*(*p*) is approximately the total rate at which demographic events are occurring and so is a measure of the rate of population turnover. Formally, it is also the variance in per-capita growth rate at selective neutrality.

Using a diffusion approximation of the full, individual-based, stochastic process [23, 24] (see S1 Appendix) and eliminating the fast timescale dynamics [25–28], the evolutionary change in frequency of social actors in the population is described by the stochastic differential equation (SDE)

$$dp=\alpha (p)dt+\sqrt{\sigma^{2}(p)}dW_{t}$$ (1)

where *α*(*p*) ≡ *μ*(1 − 2*p*) − *ϵc*(*p*)*p*(1 − *p*), *σ*^2^(*p*) ≡ *p*(1 − *p*)*T*(*p*)/[Ω*n*(*p*)], *W*_*t*_ is a Wiener process and we have neglected terms of order *ϵ*/Ω and *μ*/Ω (see S1 Appendix). Eq 1 is associated with a one-dimensional diffusion process with infinitesimal mean and variance *α*(*p*) and *σ*^2^(*p*) [29, 30]; when written as an SDE, the expression *α*(*p*)*dt* is often referred to as the “drift term”. If mutation rate is sufficiently large, the diffusion process admits a stationary distribution, which we will denote by *π*(*p*).

Note that in contrast to previous work (e.g., [16]), here our focus is the frequency of the social trait, *p*, rather than the density of social actors, *n*(*p*)*p*. As a consequence, there are no noise-induced effects in the drift term of Eq 1, whereas there are often noise-induced effects in the drift term of the SDE describing the change in density of social actors (see S1 Appendix, and also [16]). We opt to focus upon the frequency SDE rather then the density SDE because we are concerned with evolutionary processes, and evolution is a change in frequency not density.

## Results

We wish to use Eq 1 to determine if stochasticity favours one type over another. Since *α*(*p*)*dt* represents the expected change in frequency of the social actors, while $\sqrt{\sigma^{2}\left(p\right)}dW_{t}$ represents stochastic noise around this mean change, one is tempted to simply examine the sign of *α*(*p*). With this approach, if *α*(*p*) < 0 then the social actor (type 1) is disfavoured, which is the same conclusion as the deterministic model (Ω → ∞), and so this approach fails to take into account the role played by stochasticity. A second approach would be to suppose that whenever a mutation arises, it is either lost or sweeps to fixation before another mutation occurs, and so evolution proceeds according to a mutation-fixation process [31, 32]. With this approach, assessing if a trait is favoured or not is often done by comparing the probability a trait *i* mutant sweeps to fixation in a population monomorphic for trait *j* to the role-reversed situation (a comparison of invasion probabilities). If the costs of the social behaviour due to selection are sufficiently weak, *ϵ* ≈ 0, then from Eq 1 the invasion probability of a single social actor in a population of non-social individuals is 1/[Ω*n*(0)], whereas the invasion probability of a single non-social individual in a population of social actors is 1/[Ω*n*(1)]. Hence a comparison of invasion probabilities favours the social actor whenever the social trait increases population size (altruism) [14–16]. The problem with comparing invasion probabilities alone is doing so fails to consider the full evolutionary process. Because in a mutation-fixation process the population transitions from monomorphic state to monomorphic state, we can construct a Markov chain on the space of possible traits by letting *N*_*i*_ be the size of a trait-*i* population and *μ*_*ij*_ be the per-capita rate at which trait *i* mutates to trait *j*. Then the population will transition from a trait *i* state to a trait *j* state at a rate *μ*_*ij*_*N*_*i*_ × (1/*N*_*i*_) = *μ*_*ij*_. Thus in the absence of any biases in per-capita mutation rate, the population is equally likely to be observed in any monomorphic state, irrespective of the effect the trait has upon population size [32–34].

What both of these approaches have failed to take into account is the speed at which the change in population composition (and hence the evolutionary process) occurs. In particular, although the stochastic noise does not induce an average directionality to the change in *p*, the amount of stochasticity nevertheless is typically different for different values of *p*, and this will effect the speed at which the population composition changes, affecting the likelihood of observing the process in a particular state. As an analogy, a biased random walk whose step-size and time between steps depends upon the position of the walker will tend to spend more time in regions with smaller step-sizes and less frequent steps, independent of any bias in the directionality of the walk. Thus we will say that the social actor is favoured if, in the long-term, we are more likely to observe the system in a state in which the social actor is at greater frequency than the non-social actor (see S1 Appendix). For example, in the case where a stationary distribution *π*(*p*) exists, the social actor is favoured if $\int_{1/2}^{1}\pi \left(p\right)dp>1/2$.

To understand how this applies to the stochastic process defined by Eq 1, first suppose the social trait is cost-free (*ϵ* = 0). Then the behaviour of Eq 1 is determined by two factors: the magnitude of the mutation rate *μ* and the ratio *T*(*p*)/*n*(*p*). Mutation does not directly favour one type over the other and therefore the ratio *T*(*p*)/*n*(*p*) should play a critical role in determining the values of *p* at which the system spends the most time. The following derivative tells us how this ratio changes with *p*:

$$\frac{d}{dp}\left[\frac{T(p)}{n(p)}\right]=\frac{T(p)}{n(p)}(\underset{\left(\text{i}\right)}{\underset{︸}{-\frac{dn/dp}{n(p)}}}+\underset{\left(\text{ii}\right)}{\underset{︸}{\frac{dT/dp}{T(p)}}}).$$ (2)

There are two components to Eq 2, each with a simple biological interpretation: (i) is the effect the social trait has upon population size, *n*(*p*), and (ii) is the effect the social trait has upon population turnover, *T*(*p*). In terms of our random walk analogy, as the population size increases, the step size of the random walk (in terms of frequency *p*) decreases, meaning that the process will tend to spend more time at values of *p* corresponding to large population sizes. Put another way, larger populations are more buffered against demographic stochasticity and thus effect (i) shows how the type resulting in the greatest population size tends to be favoured [14–16]. Likewise, the rate of population turnover (as measured by the neutral variance in per-capita growth rate, *T*(*p*)) can be thought of as controlling the frequency of steps taken by the random walker. Thus the process will tend to spend more time at values of *p* that correspond to less frequent steps, and so effect (ii) shows how the type minimizing *T*(*p*) tends to be favoured. Taken together these two effects therefore favour the type minimizing the amount of demographic stochasticity, as given by the ratio *T*(*p*)/*n*(*p*).

We can now examine how the different social traits influence effects (i) and (ii). If the social trait is altruism, then as explained earlier the population size will increase as its frequency increases (i.e., *dn*/*dp* > 0; this process was the focus of previous work on the role of demographic stochasticity [14–16]). On the other hand, if the trait is spite then the population size will decrease as its frequency increases (i.e., *dn*/*dp* < 0). Thus effect (i) always favours altruism and disfavours spite. The role played by effect (ii) is more complex. To see why, observe that on the slow timescale the demographic processes are in quasi-equilibrium and so *T*(*p*) = 2*b*(*p*) = 2*m*(*p*). Therefore if either *b*(*p*) or *m*(*p*) are constant with respect to *p* then *dT*/*dp* = 0. In this case only term (i) plays a role and so altruism is always favoured and spite disfavoured. Otherwise, to understand how the social action affects *T*(*p*), we need to consider two cases: (a) the social action affects the death rate, or (b) the social action affects the birth rate.

Consider the case where the social action affects the death rate. If the the social action is altruism then by definition it must decrease the death rate (*dm*/*dp* < 0) and so we have *dT*/*dp* < 0. Conversely, if the social action is spite then by definition it must increase the death rate (*dm*/*dp* > 0) and so *dT*/*dp* > 0. In both cases effect (ii) works in concert with effect (i) to always favour altruism and disfavour spite. Indeed the ratio *T*(*p*)/*n*(*p*) is monotonic is *p*, being minimized at *p* = 1 in the case of altruism and at *p* = 0 in the case of spite.

Next consider the case where the social action affects the birth rate. If the social action is altruism then by definition it must increase the birth rate (*db*/*dp* > 0) and so we have *dT*/*dp* > 0. On the other hand, if the social action is spite then by definition it must decrease the birth rate (*db*/*dp* < 0) and so we have *dT*/*dp* < 0. Hence effect (ii) opposes effect (i). As a result, altruism or spite can each be favoured or not depending upon the magnitude of effect (i) relative to the magnitude of effect (ii). Moreover, the ratio *T*(*p*)/*n*(*p*) can be non-monotonic, meaning that it can be minimized by a polymorphic population.

To illustrate these phenomena more concretely, we apply our analysis to several specific models (see S1 Appendix for details). Throughout we use *x*_*i*_ to denote the density of type *i*.

**Fig 1:**
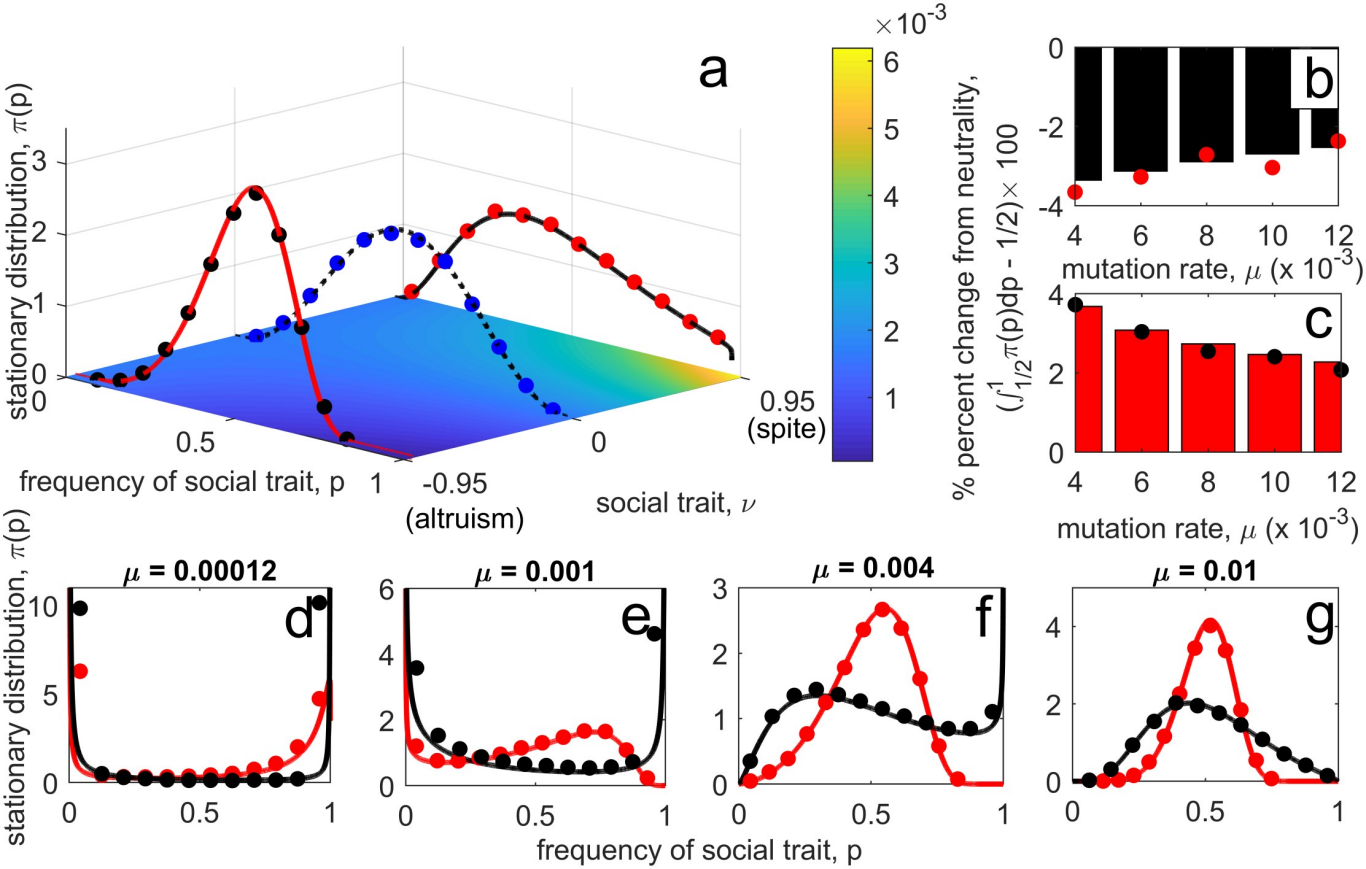
Role of demographic stochasticity in the evolution of cost-free social traits acting on death rate. The model uses *b* ≡ 3(1 − *x*_1_ − *x*_2_), *m* ≡ 1 + *νx*_1_/(*x*_1_ + *x*_2_) with Ω = 900; if *ν* > 0, the trait is spite, whereas if *ν* < 0, the trait is altruism. Subplot **a**—stationary distributions corresponding to three different values of *ν*: altruism (*ν* = −0.95), neutral (*ν* = 0), and spite (*ν* = 0.95), revealing the close match between our analytic results and simulations of the full stochastic process. Mutation rate is *μ* = 0.006 in all cases. The distribution is skewed towards a higher frequency of the social actor in the case of altruism and towards a lower frequency of the social actor in the case of spite (distribution is symmetric in the neutral case). Underlying contour plot shows the value of the ratio *T*(*p*)/[Ω*n*(*p*)]. Subplots **b** and **c** shows the degree to which the social actor is disfavoured (spite, *ν* = 0.95; subplot **b**) or favoured (altruism, *ν* = −0.95; subplot **c**) for different mutation rates: as mutation rate decreases, the effect of demographic stochasticity increases. Subplots **d**-**g** show how changing mutation rate alters the shape of the stationary distribution. When mutations are low (subplot **d**), the stationary measure is *U*-shaped, but skewed in favour of the social actor if the trait is altruism (red curve) or non-social actor if the trait is spite (black curve). As mutation rate increases, the distribution is initially pushed into the interior at the boundary for which the ratio *T*(*p*)/*n*(*p*) is minimized (*p* = 1 and red curve on subplot **e**; *p* = 0 and black curve on subplot **f**), before the distribution ultimately becomes unimodal with distribution favouring the type which minimizes *T*(*p*)/*n*(*p*) (subplot **g**). In all plots, the curves/bars are analytic predictions, while circles are the average of 3 × 10^4^ simulations of the full stochastic process (see S1 Appendix). For subplot **b** and **c**, simulations were terminated after 5 × 10^5^ and 7.5 × 10^5^ time units, respectively.

**Fig 2:**
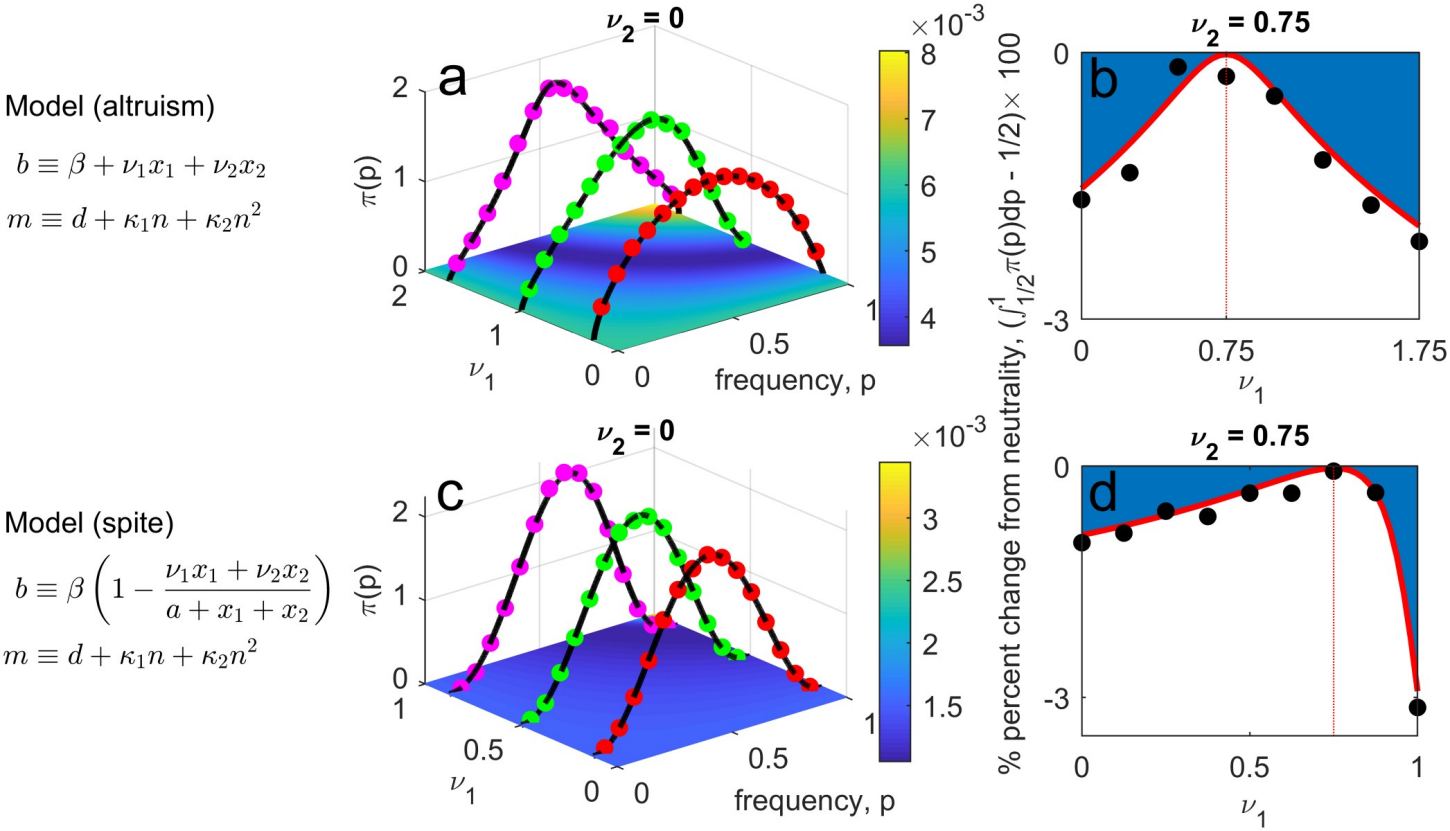
Role of demographic stochasticity in the evolution of cost-free social traits acting on birth rate. In subplots **a**-**b**, the social trait is altruism. In subplots **c**-**d**, the trait is spite. Subplot **a**—stationary distributions corresponding to three different strengths of altruism *ν*, showing how altruism can be disfavoured. Subplot **c**—stationary distributions corresponding to three different strengths of spite *ν*, showing how spite can be favoured. Underlying contour plot shows the value of the ratio *T*(*p*)/[Ω*n*(*p*)]. Subplots **b,d** show that in some cases an intermediate level of social action is optimal. Subplot **b**—a type that uses the intermediate level of altruism that minimizes the ratio *T*(*p*)/*n*(*p*) in a monomorphic population (in this case *ν* = 0.75) is favoured over all other levels of altruism. Subplot **d**—a type that uses the intermediate level of spite that minimizes the ratio *T*(*p*)/*n*(*p*) in a monomorphic population (in this case *ν* = 0.75) is favoured over all other levels of spite. Curves are analytic predictions and each circle is 6 × 10^4^ simulations of the full stochastic process; simulations were run for 10^3^ and 5 × 10^4^ time units for subplots **b** and **d** respectively. Parameters values: {*β*, *d*, *κ*_1_, *κ*_2_, Ω, *μ*} = {1, 0.5, 0.75, 0.01, 250, 0.01} (subplots **a**-**b**) and {*β*, *d*, *a*, *κ*_1_, *κ*_2_, Ω, *μ*} = {8, 1, 0.05, 0.05, 0.2, 900, 0.005} (subplots **c**-**d**).

1. **Social action alters death rate.** Consider a population in which social actors alter the death rate of others. This could be through, for example, the actors producing a diffusible compound such as a resource (e.g., the enzyme invertase in *S. cerevisiae* [16, 19]) or toxin (e.g., bacteriocins [20]). Let *m* ≡ *d*(1 + *νx*_1_/[*x*_1_ + *x*_2_]), with *ν* ∈ (−1, 1). Then the type of social trait is determined by the sign of *ν*: if *ν* > 0, the trait is spite, whereas if *ν* < 0 the trait is altruism. Suppose population size is regulated by density-dependent fecundity, and so let *b* ≡ *β*(1 − *x*_1_ − *x*_2_), with *β* > *d*(1 + |*ν*|). Thus *T*(*p*)/*n*(*p*) is decreasing in *p* if *ν* < 0 (altruism) and increasing if *ν* > 0 (spite) (see also S1 Appendix). Fig 1 shows that, as our analysis predicts, demographic stochasticity favours altruism and disfavours spite. It also illustrates how the evolutionary outcome depends upon mutation rate.
2. **Social action alters birth rate.** Here we consider separate models for altruism and spite. For altruism, we suppose that social actors produce a public good that increases growth/reproduction, and that uptake of this good occurs through mass-action contact between the social actor and the recipient. One such example is the production of siderophores by *Pseudomonas aeruginosa* to scavenge iron essential for bacterial growth [35]. As such, we suppose social actors increase the birth rate of others by an amount *ν* > 0, and so let *b* ≡ *β* + *νx*_1_. For spite, suppose individuals attempt to reproduce at a per-capita rate *β*, and with probability *νx*_1_/(*x*_1_ + *x*_2_ + *a*) reproduction is blocked by a social actor (so *ν* ∈ [0, 1], *a* > 0), and thus *b* ≡ *β*(1 − *νx*_1_/[*x*_1_ + *x*_2_ + *a*]). This could represent a population of sexual hermaphrodites such that when social actors play the role of ‘male’ they spitefully reduce their investment in gametes, leading to reproductive failure (so *a* controls probability of self-fertilization). For both models, let the per-capita mortality rate be *m* ≡ *d* + *κ*_1_(*x*_1_ + *x*_2_) + *κ*_2_(*x*_1_ + *x*_2_)^2^. Fig 2a and 2c shows that, as our analysis predicts, demographic stochasticity can now disfavour altruism and favour spite. In fact, the ratio *T*(*p*)/*n*(*p*) can be non-monotonic in *p* and so be minimized by a polymorphic population containing social actors and non-actors. This also suggests that in such cases an intermediate level of social action might, in some sense, be optimal (see S1 Appendix). For example, in a monomorphic population the value of *ν* minimizing the ratio *T*/*n* for the altruism model is ν* = *κ*_1_ − |*β* − 2*d*|/*θ*, where $\theta =\sqrt{d/\kappa_{2}}$, and for the spite model is ν* = (*a* + *θ*)(*β* − 2*d* − *κ*_1_*θ*)/(*βθ*). Fig 2b and 2d shows that when one type of actor displays this level of social action, all other levels of social action *ν* are disfavoured.

**Fig 3:**
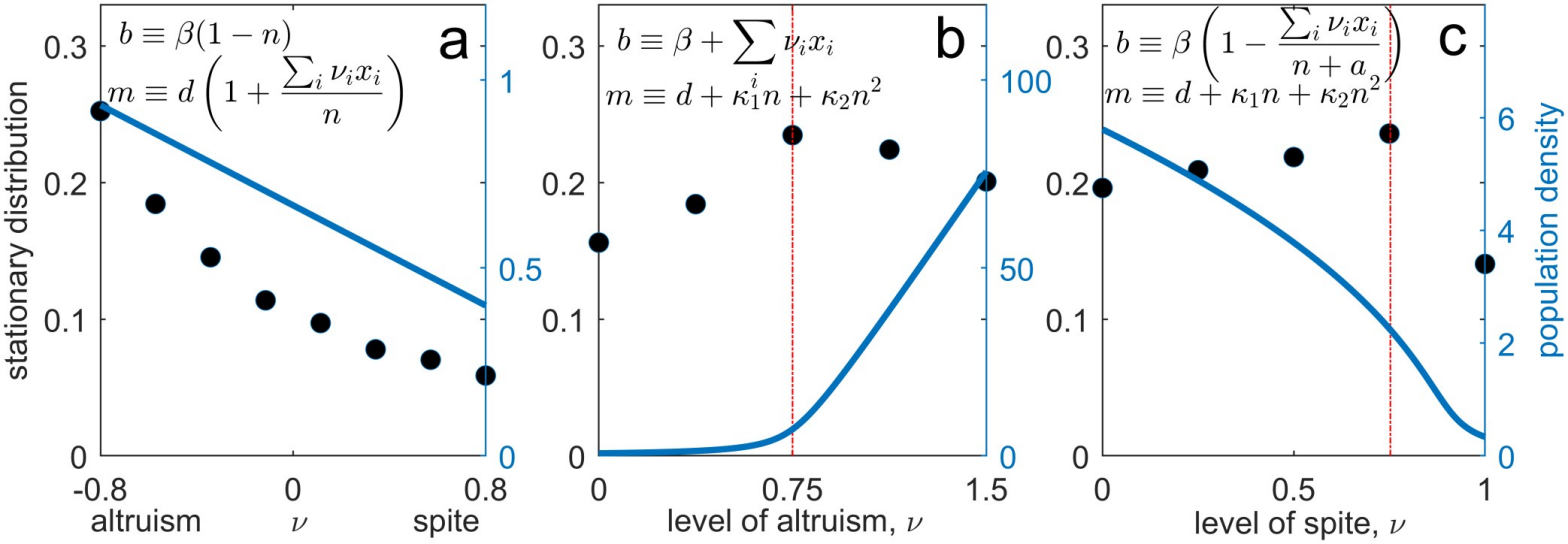
Role of demographic stochasticity in the evolution of cost-free social traits. Each subplot is the model indicated by the per-capita birth and death rates, *b* and *m*, with *n* = ∑_*i*_ *x*_*i*_. The black circles are the results of 10^4^ simulations of the system of SDEs (S1 Appendix) and represent the probability of observing the simulation in a given state (left *y*-axis). The blue curve is the expected population density of a population monomorphic for the trait value (right *y*-axis). If population size alone was sufficient to predict which trait is favoured, we would expect a close match between the stationary distribution (black circles) and population size (blue curve)—this does not occur because what is important is the ratio *T*/*n*. Indeed, as predicted by consideration of (1), the stochastically favoured trait for subplot **a** is altruism, whereas for subplot **b** and **c** it is the trait value at the red dashed line. Parameter values used: subplot **a**, {*β*, *d*} = {3, 1}, subplot **b**, {*β*, *d*, *κ*_1_, *κ*_2_} = {1, 0.5, 0.75, 0.01}, and subplot **c**, {*β*, *d*, *κ*_1_, *κ*_2_, *a*} = {8, 1, 0.05, 0.2, 0.05}. All simulations used Ω = 10^4^ and assumed type *i* mutates to type *j* at a per-capita rate *μ* = 10^−6^.

Simulation results suggest that when more than two types of individuals are included in the population, the above results hold. For example, Fig 3a shows that when the social trait acts on death rate and there are several different types of individuals in the population, ranging from very altruistic to very spiteful, it is the most altruistic type that is favoured. Furthermore, Fig 3b and 3c shows that when the social trait acts on birth rate and there are multiple types of individuals in the population, it can be an intermediate level of altruism or spite that is favoured (analogous to Fig 2b and 2d). Up until this point we have assumed the social trait is cost-free, *ϵ* = 0. Suppose instead the social action has a cost, *ϵ* > 0, which creates a directional bias disfavouring the social trait. We may then ask if/when the effect of stochastic noise can overcome this directional bias, and so reverse the direction of selection [14–16]. We will focus upon situations in which a stationary distribution, *π*(*p*), exists. Since by construction the social actor (type 1) is at a selective disadvantage (*ϵ* > 0), if $\int_{1/2}^{1}\pi \left(p\right)dp>1/2$, then we may argue demographic stochasticity reverses the direction of selection.

We illustrate this phenomenon with two examples. First, consider a population where the social actor is an altruist capable of altering birth rate such that *b* ≡ *r* + *νx*_1_, *m* ≡ *κ*(*x*_1_ + *x*_2_), and *c* ≡ *r*, where *r* > 0, *κ* > *ν* > 0. Models based on these specific assumptions have been explored by previous authors, where it was argued that demographic stochasticity favours altruism and thus a selective reversal is possible [14–16]. This argument was based upon two main points. First, the authors observed that the drift term of the SDE associated with the density of social actors, *pn*(*p*), could be either positive or negative due to the magnitude of noise-induced effects relative to selection. Second, the authors showed that whichever phenotype can grow to a larger population size in isolation is favoured (altruists) by applying a pairwise comparison of invasion probabilities. Each of these points has an interpretative issue. First, although noise-induced effects often appear in the drift term of the SDE describing the change in density of social actors (the ecological process), these tend to disappear after the density SDE is converted to the SDE tracking the frequency of the social trait (the evolutionary process), and this is indeed the case here (see Eq 1). It is these noise-induced effects that lead to the incorrect conclusion about when social traits are favoured. To see why, consider the above model when there are no mutations, *μ* = 0, and no selection, *ϵ* = 0. Then the social trait (altruism) is neutral. Suppose the population is initially at a state in which half the individuals are social actors, *p* = 1/2. Then since the fixation probability of the social actor in a neutral population is equal to its proportion in the population, 50% of the time the social actor will sweep to fixation in the population. Unsurprisingly, the drift term for the frequency equation, *α*(*p*), in Eq 1 is zero, that is, the expected change in frequency is zero. However, the drift term of the SDE for the density of social actors will be positive. This is because the population size goes up when the altruists fix more than it goes down when non-altruists fix. But altruism is neutral, and therefore the sign of the drift term of the density equation cannot be used as a measure of evolutionary ‘success’. Second, although comparison of invasion probabilities does favour whichever phenotype grows to a larger population size in isolation, as we pointed out previously, if we place the invasion probabilities within the context of the full mutation-fixation evolutionary process the effect of population size disappears (see also [32–34]).

**Fig 4:**
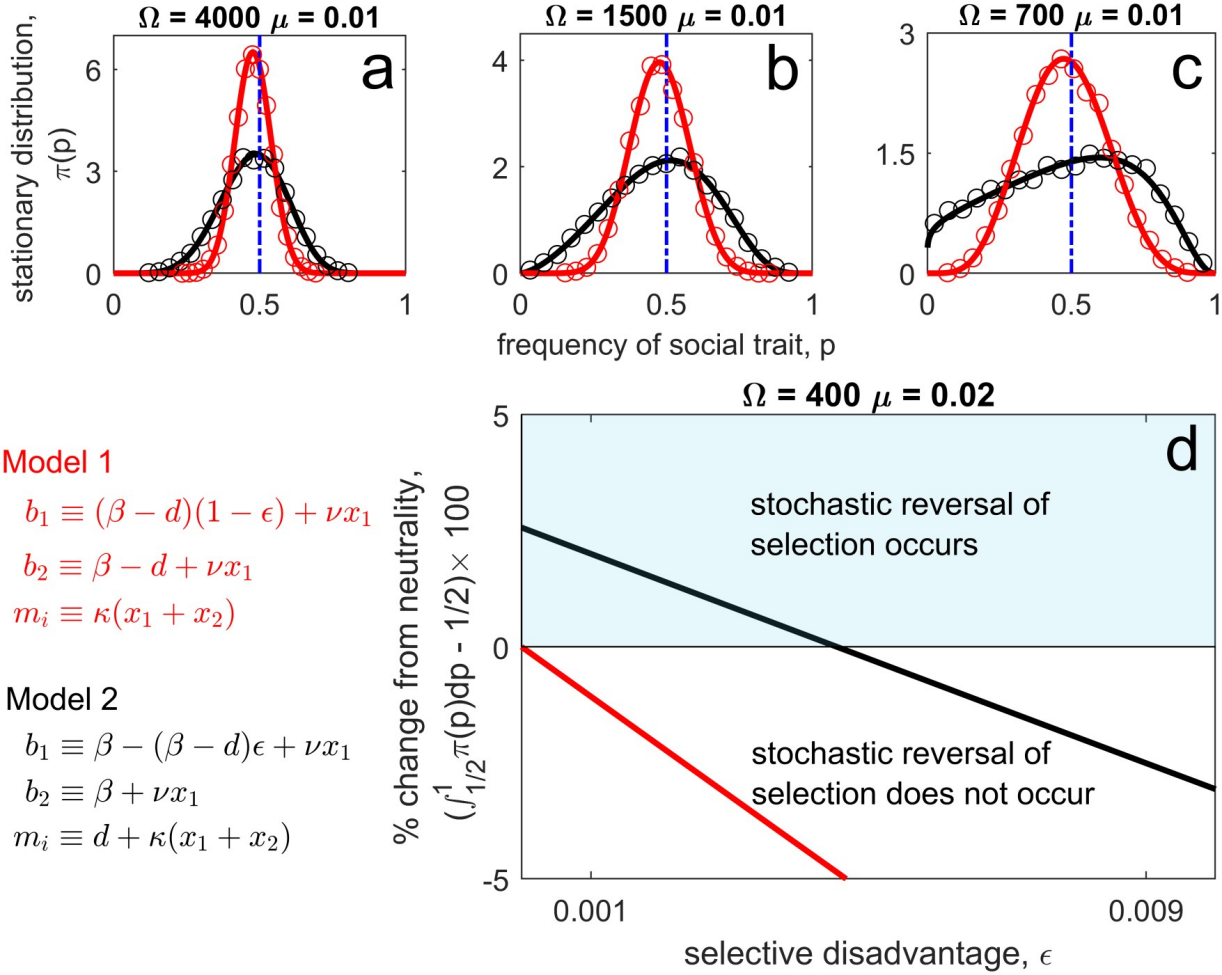
Can stochasticity reverse selection? Here we compare two models of altruism having the same per-capita growth rate, but differing in variance in per-capita growth (Model 1-red, Model 2-black). As a consequence, this can lead to a stochastic reversal of selection for model 2 but not model 1. Subplots **a**-**c**: predicted stationary distribution for both models from Eq 1 (curve) compared to 2 × 10^4^ simulations of the full stochastic process (circles) for decreasing habitat size, Ω (i.e., increasing levels of demographic stochasticity). The distribution is always skewed towards the non-altruist in model 1. For model 2 the distribution changes from being skewed towards the non-altruist to being skewed towards the altruist as demographic stochastic increases (i.e., a selective reversal occurs). Subplot **d**: magnitude of the selective reversal plotted against the cost of altruism. Parameters used were {*β*, *d*, *κ*, *ν*} = {3, 2.4, 1.2, 1}, with *ϵ* = 0.003 for subplots **a**-**c**.

Indeed, these issues can be made readily apparent by considering the stationary distribution associated with the model (this assumes mutations are explicitly included, which deviates from the model in [16]). In particular, the stationary distribution is

$$\pi (p)\propto p^{\frac{\mu \Omega }{\kappa }-1}{\left(1-p\right)}^{\frac{\mu \Omega }{\kappa }-1}e^{-\frac{\epsilon r\Omega }{\kappa }p},$$ (3)

(see S1 Appendix). If *μ*Ω/*κ* > 1, then mutations push the distribution towards *p* = 1/2 and so *π*(*p*) has a (skewed) bell-shape, whereas if *μΩ*/*κ* < 1, the distribution accumulates at *p* = 0 and *p* = 1 and so *π*(*p*) has a (skewed) U-shape. At selective neutrality, *π*(*p*) is symmetric about *p* = 1/2 and so altruism is completely neutral. If altruism comes at a cost, *ϵ* > 0, then *π*(*p*) is shifted in favour of the non-actor and so stochasticity can never reverse the direction of selection (Fig 4, Model 1—red). This conclusion can also be reached by noting the ratio *T*(*p*)/*n*(*p*) in this particular model is a constant, independent of *p* (S1 Appendix). This is because any increase in population size (which reduces the step size of the random walk in *p*) is exactly compensated for by an increase in the rate of population turnover. Interestingly, it is possible to construct a model in which a selective reversal occurs by making only a slight modification of the above assumptions. Suppose *b* ≡ *β* + *νx*_1_, *m* ≡ *d* + *κ*(*x*_1_ + *x*_2_), and *c* ≡ *r*, with *r* = *β* − *d*. This model has the same per-capita growth rate as the previous model but now the rate of population turnover (i.e., the variance in per-capita growth) is larger. As a result, the ratio *T*(*p*)/*n*(*p*) is linearly decreasing in *p*. The stationary distribution is then

$$\pi (p)\propto p^{\frac{\mu \Omega r}{\beta \kappa }-1}{\left(1-p\right)}^{\frac{\mu \Omega r}{\beta \kappa -d\nu }-1}{\left(\beta \kappa -d\nu p\right)}^{\frac{r^{2}\Omega \epsilon }{d\nu }-\frac{\mu \Omega r}{\beta \kappa }-\frac{\mu \Omega r}{\beta \kappa -d\nu }-1}.$$ (4)

In this modified model altruism is now stochastically favoured (Fig 4, Model 2—black) and so stochasticity can reverse the direction of selection. Notice from Eq 4 the role played by mutation rate in shaping the stationary distribution. In the first model, mutation rate only controlled whether the distribution was normalizable or not. Now, however, mutation rate can alter whether or not a selective reversal is possible.

It is important to stress that the difference in outcome between these two models is driven exclusively by demographic stochasticity. The deterministic components of these two models are the same. Put another way, the expected change in the frequency of the altruists is identical in the two models despite the second model predicting the evolution of costly altruism while the first model not doing so. In the first model selection pushes the distribution in favour of the non-altruists and demographic stochasticity has no biasing effect. In the second model, again selection pushes the distribution in favour of the non-altruists, but now demographic stochasticity is biased such that it decreases as the altruists become more common. The predicted population composition (i.e., the stationary distribution) thus arises from a balance between selection favouring non-altruists and the demographic noise being smaller when the frequency of altruists is high. These effects only become apparent from consideration of the ratio *T*(*p*)/*n*(*p*). Thus determining whether stochasticity can reverse selection requires analysis of this ratio, and we cannot exclusively focus upon how the social trait alters population size [14–16].

## Discussion

Recent work has explored how stochasticity can alter social trait evolution by deriving a stochastic version of Hamilton’s rule [17]. Our work differs from this in a couple of important ways. First, those authors focused upon the expected evolutionary change alone, which is equivalent to considering the sign of *α*(*p*) of Eq 1, whereas our focus is upon how social traits influence the evolutionary noise, and how this works in conjunction with the expected evolutionary change. Our results demonstrate that examining the expected evolutionary change alone may often be insufficient to determine whether a social trait subject to stochasticity is more or less likely to be observed. Instead one may need to account for both the expected change in the population composition as well as any change in (unbiased) demographic noise that occurs during evolution (i.e., the ratio *T*(*p*)/*n*(*p*)). Second, we have focused on indiscriminate social behaviours and as such, in well-mixed populations these traits are always either neutral (if they are cost-free) or selected against (if they entail a cost). In contrast, Kennedy et al. [17] focuses upon cooperation preferentially directed towards kin.

Our analysis has focused upon unstructured populations in which every individual is equally likely to interact with every other individual. It is well known that population structure can aid or hinder the evolution of social traits [5–7, 36–38] by altering the likelihood that similar or dissimilar social actors interact with one another. Demographic stochasticity will likely factor into this (see in particular [16]), but its impact will depend upon the relatedness of interacting individuals as well as the magnitude of the benefits of the social trait. As relatedness between individuals increases, in general so too will the strength of selection (by generating indirect fitness benefits), which will tend to diminish the role of demographic stochasticity. However, in populations with low relatedness, or social behaviours with sufficiently low benefits (and costs), we would expect our theory to apply. Interestingly, as shown in [16], in deme-structured populations although the social behaviour can be disfavoured or neutral at the within-deme level, it can be favoured at the between-deme level if the social behaviour increases population size and so the number of dispersers [16].

An interesting parallel to our results is that in structured populations, helping behaviours effecting fecundity tend to be selectively favoured over those which effect survivorship [36–39]; a prediction that diverges from our model. One key difference between our model and these previous studies is that they focused upon the expected change in the social trait in populations of fixed size; as such, whether the helping behaviour is interpreted as one which effects survivorship or one which effects fecundity is based upon whether the population evolves through birth-death or death-birth updating. Hence this result is mediated through the scale of competition between interactants, whereas our result occurs through how the social action effects the evolutionary noise the population experiences.

The role played by demographic stochasticity in populations of fluctuating size has received increased attention recently [14–16, 40, 41]. Our work here has provided a very general consideration of the evolution of two fundamental social traits, altruism and spite, and this analysis has revealed the importance of the action of the social trait upon the recipient. In particular, if the social action alters death rate, then provided selection is sufficiently weak, altruism is stochastically favoured while spite is stochastically disfavoured. If instead the social action alters birth rate, altruism and spite can be either favoured or disfavoured, depending upon mutation rate, the underlying population demography and how this determines the ratio of the rate of population turnover to the population size, *T*(*p*)/*n*(*p*). The generality of our analysis suggests this principle likely has implications across other study systems as well.

## Supporting information

S1 Appendix Supplementary information. Full derivation of model and details of mathematical analysis.
(PDF)
