# Supplementary material for: Social evolution under demographic stochasticity
Source: PLoS Comput Biol. 2019 Feb 4;15(2):e1006739. doi: 10.1371/journal.pcbi.1006739 (PMC6375627; doi:10.1371/journal.pcbi.1006739)
Supplement: S1 Appendix — Full derivation of model and details of mathematical analysis. (PDF) [file pcbi.1006739.s001.pdf]

Supporting information for "Social evolution under demographic stochasticity"

David V. McLeod[1*], Troy Day[2†]

1 Institute for Integrative Biology, ETH Zürich, Zürich, Switzerland
2 Department of Mathematics and Statistics, Department of Biology Queen's University, Kingston, ON, Canada

* david.mcleod@env.ethz.ch
† day@queensu.ca

General model

We first derive the general population model by specifying a continuous time discrete state space Markov process. To do so, consider a population consisting of two types of individuals and potentially some environmental variable that the two types interact with (e.g., a diffusible compound produced by type 1). Let X_i denote the number of individuals of type $i = 1, 2$ at time t and Y denote the state of the environmental variable. Let $\mathbf{X} = (X_1, X_2, Y)$. The infinitesimal transition probabilities regulating the population demographics are

$$\begin{array}{llll} \text{Event type} & \text{Transition} & \text{Probability} & \\ \text{birth of type } i & \mathbf{X} \to \mathbf{X} + \mathbf{e}_i & \mathbb{T}_i(\mathbf{X}) \equiv b_i(\mathbf{X}) X_i \Delta t, & i, j = 1, 2, \\ \text{death of type } i & \mathbf{X} \to \mathbf{X} - \mathbf{e}_i & \mathbb{T}_{i+2}(\mathbf{X}) \equiv m_i(\mathbf{X}) X_i \Delta t, & i \neq j \\ \text{mutation of type } i \text{ to } j & \mathbf{X} \to \mathbf{X} - \mathbf{e}_i + \mathbf{e}_j & \mathbb{T}_{i+4}(\mathbf{X}) \equiv \mu X_i \Delta t & \end{array} \tag{S1}$$

where $\mathbf{e}_i$ is the 1×3 vector with a 1 in the i-th spot and zeros in the others. We do not explicitly specify the dynamics of the environmental variable for reasons which will become apparent shortly, but suppose that it may under go any possible transition of the form $\mathbf{X} \to \mathbf{X} \pm \mathbf{e}_3$ or $\mathbf{X} \to \mathbf{X} \pm \mathbf{e}_3 \mp \mathbf{e}_i$. In the latter case, the effect upon type 1 or 2 is subsumed into the birth and death terms of eq S1. Let $Q(\mathbf{X}, t)$ be the probability density for $\mathbf{X}$ at time t. Then the master equation for the stochastic process (ignoring changes in Y) is

$$\begin{aligned} \frac{\partial Q}{\partial t} = \sum_{i=1}^{2} \Big[\mathbb{T}_i(\mathbf{X} - \mathbf{e}_i) Q(\mathbf{X} - \mathbf{e}_i, t) + \mathbb{T}_{i+2}(\mathbf{X} + \mathbf{e}_i) Q(\mathbf{X} + \mathbf{e}_i, t) \Big] \\ + \sum_{\substack{i,j=1,2 \\ i \neq j}} \mathbb{T}_{i+4}(\mathbf{X} + \mathbf{e}_i - \mathbf{e}_j) Q(\mathbf{X} + \mathbf{e}_i - \mathbf{e}_j, t) - \sum_{k=1}^{6} \mathbb{T}_k(\mathbf{X}) Q(\mathbf{X}, t). \end{aligned} \tag{S2}$$

Let Ω be a system size parameter (e.g., [1, 2]), and take $\mathbf{x} \equiv (x_1, x_2, y) \equiv \mathbf{X}/\Omega$, and $q(\mathbf{x}, t) \equiv \Omega Q(\mathbf{X}, t)$. In our context Ω can be thought of as habitat size and so $\mathbf{x}$ can be thought of as densities. If we suppose Ω is sufficiently large such that the variables $\mathbf{x}$ are approximately continuous and rescale time as $\tau = t/\Omega$, we can perform a series expansion in powers of $1/\Omega$ to give the Fokker-Planck (or forward Kolmogorov) equation

$$\frac{\partial q}{\partial \tau} = -\sum_{i=1}^{2} \frac{\partial}{\partial x_i} A_i(\mathbf{x}) q(\mathbf{x}, \tau) + \frac{1}{2\Omega} \sum_{i=1}^{2} \sum_{j=1}^{2} \frac{\partial^2}{\partial x_i x_j} B_{ij}(\mathbf{x}) q(\mathbf{x}, \tau) \tag{S3}$$

where $A_i(\mathbf{x}) = (b_i(\mathbf{x}) - m_i(\mathbf{x}))x_i - \mu(x_i - x_j)$, and the $B_{ij}(\mathbf{x})$ are the entries of

$$\boldsymbol{B}(\mathbf{x}) = \begin{pmatrix} (b_1(\mathbf{x}) + m_1(\mathbf{x}))x_1 + \mu n & -\mu n \\ -\mu n & (b_2(\mathbf{x}) + m_2(\mathbf{x}))x_2 + \mu n \end{pmatrix}, \tag{S4}$$

with $n = x_1 + x_2$.

The Fokker-Planck equation given by eq S3 is associated with the system of Ito stochastic differential equations (SDEs)

$$d\mathbf{x} = \boldsymbol{A}(\mathbf{x}) d\tau + \Omega^{-1/2} \boldsymbol{C}(\mathbf{x}) d\mathbf{W}_\tau \tag{S5}$$

where $\boldsymbol{A}(\mathbf{x}) = (A_1(\mathbf{x}), A_2(\mathbf{x}))^T$, $\boldsymbol{C}(\mathbf{x})\boldsymbol{C}(\mathbf{x})^T = \boldsymbol{B}(\mathbf{x})$ and $\mathbf{W}_\tau$ is a vector of N independent Wiener processes [1, 3]. Thus $\boldsymbol{C}(\mathbf{x})$ is a $2 \times N$ matrix, where N is a integer whose value will depend upon how the matrix $\boldsymbol{C}(\mathbf{x})$ is chosen (the choice of matrix $\boldsymbol{C}(\mathbf{x})$ is not

unique). If habitat size becomes large, $\Omega \to \infty$, stochasticity disappears from eq S5, and we are left with the system of ordinary differential equations (ODEs) $\dot{\mathbf{x}} = \boldsymbol{A}(\mathbf{x})$, where $\dot{\mathbf{x}} = (dx_1/d\tau, dx_2/d\tau)^T$ (we have purposefully neglected $dy/d\tau$ in $\dot{\mathbf{x}}$).

As in the main text, we assume that type 1 is the social actor, and so if we let $r_i(\mathbf{x}) \equiv b_i(\mathbf{x}) - m_i(\mathbf{x})$ denote the per-capita growth rate of type i, then we assume that $r_1(\mathbf{x}) = b(\mathbf{x}) - m(\mathbf{x}) - \epsilon c(\mathbf{x})$ and $r_2(\mathbf{x}) = b(\mathbf{x}) - m(\mathbf{x})$. Thus whenever $\epsilon > 0$, type 2 has the selective advantage, and will ultimately fix in the ODE model without mutations. Note that the costs may either reduce birth rate or increase death rate; although in general this will have implications for the structure of $\boldsymbol{B}(\mathbf{x})$ and thus $\boldsymbol{C}(\mathbf{x})$, because we will assume ϵ is sufficiently small that we can neglect terms of order ϵ/Ω, we only need to take into account how the costs alter expected per-capita growth rate.

Reduction of system to slow manifold

We wish to reduce system S5 into a more manageable problem. To do so, suppose that in the absence of selection, mutations, and stochasticity ($\epsilon = 0$, $\mu = 0$, and $\Omega \to \infty$, respectively), there exists a globally asymptotically stable curve of ecological equilibria in the ODE system given by $r_i(\mathbf{x}) = 0$. We parameterize this curve in terms of x_1, and so let $\boldsymbol{\gamma}(w) = (w, \gamma_2(w), \gamma_y(w))$ denote the value of $\mathbf{x}$ along the curve. We will assume that both $w/\gamma_2(w)$ and $w/(w + \gamma_2(w))$ are invertible. When $\mu = \epsilon = 0$ and $\Omega \to \infty$, the ODE system will asymptotically approach different points on $\boldsymbol{\gamma}(w)$ dependent upon the initial conditions ($\boldsymbol{\gamma}(w)$ is a center manifold [4]). If instead Ω is large but finite, and ϵ, μ are nonzero but small, then system S5 will rapidly approach $\boldsymbol{\gamma}(w)$ along the flow lines of the ODE system. However, once in the vicinity of $\boldsymbol{\gamma}(w)$ the stochastic component of system S5 will dominate the dynamics, since as $\mathbf{x} \to \boldsymbol{\gamma}(w)$, $\boldsymbol{A}(\mathbf{x}) \to 0$. Because the movement along $\boldsymbol{\gamma}(w)$ is slow relative to the rate at which a system initially distant moves to the vicinity of $\boldsymbol{\gamma}(w)$, in this context $\boldsymbol{\gamma}(w)$ is often referred to as a 'slow manifold' [5–7]. Since the movement along the slow manifold corresponds to change in population composition (and so represents the evolutionary timescale), our goal is to derive an equation approximating the motion of system S5 along $\boldsymbol{\gamma}(w)$.

To do so, observe that when $\epsilon = \mu = 0$ and $\Omega \to \infty$ we have

$$\frac{dx_1/d\tau}{dx_2/d\tau} = \frac{A_1(\mathbf{x})}{A_2(\mathbf{x})} = \frac{x_1}{x_2} \quad \Rightarrow \quad \frac{dx_1}{dx_2} = \frac{x_1}{x_2}, \tag{S6}$$

and so for the initial condition $\mathbf{x}_w = (w_1, w_2, w_3)$, the solution of eq S6 is $x_1(t) = (w_1/w_2)x_2(t)$. Now suppose (stochastic) trajectories along the slow manifold receive random "kicks" displacing the system from the slow manifold. Once the system is "kicked away" from the slow manifold, then provided Ω is large (and ϵ, μ small), the system will return to the slow manifold approximately along the (deterministic) flow lines. These flow lines are the solutions of eq S6 with the initial condition being the position system was "kicked" too. Thus a trajectory initially at (x_1, x_2, y) will return to the point on the slow manifold, $\boldsymbol{\gamma}(w)$, implicitly given by

$$\gamma_2(w) = \frac{x_2}{x_1} w. \tag{S7}$$

From eq S7, since w parameterizes the slow manifold, dw/dt reveals how the position of the system along the slow manifold evolves with time [6, 8]. Let $\mathcal{G}(w) = w/\gamma(w)$ and $g(w) = \mathcal{G}^{-1}(w)$. Then from eq S7 we have $w = g(x_1/x_2)$ and so applying the

multivariable version of Ito's formula [1, 3] to w gives

$$dw = \left[\sum_{i=1}^{2} \frac{\partial g}{\partial x_i} A_i(\mathbf{x}) + \frac{b(\mathbf{x}) + m(\mathbf{x})}{2\Omega} \sum_{i=1}^{2} \frac{\partial^2 g}{\partial x_i^2} x_i\right] d\tau + \sqrt{\sum_{i=1}^{2} \left(\frac{\partial g}{\partial x_i}\right)^2 \frac{(b(\mathbf{x}) + m(\mathbf{x}))x_i}{\Omega}} dW_\tau \tag{S8}$$

where we have neglected terms of order ϵ/Ω and μ/Ω.

Let $p \equiv w/(w + \gamma_2(w))$ be the frequency of type 1 along the slow manifold. Then applying Ito's formula to compute dp using eq S8 and evaluating the result on the slow manifold (see [6, 8] for justification) gives

$$dp = [\mu(1 - 2p) - \epsilon c(p)p(1 - p)]d\tau + \sqrt{\frac{p(1 - p)T(p)}{\Omega n(p)}} dW_\tau \tag{S9}$$

with $T(p) = b(\boldsymbol{\gamma}(h(p))) + m(\boldsymbol{\gamma}(h(p)))$, $c(p) = c(\boldsymbol{\gamma}(h(p)))$, $n(p) = h(p) + \gamma_2(h(p))$, where $h(p)$ is defined as $h^{-1}(w) = w/(w + \gamma_2(w))$.

To obtain eq S9 we did not need to specify the dynamics of the environmental variable y, and instead only needed to know the density of the environmental variable along the slow manifold, $\gamma_y(w)$. This is because the separation of time scales assumes population composition changes on a slower time scale than demographic processes and so on the slow timescale the environmental variable will be in a quasi-steady state if we use the tools of Parsons and Rogers [6].

As an aside, we note that for problems which satisfy condition eq S6, then eq S9 is the same result we would have obtained if we instead applied Ito's formula to $p = x_1/(x_1+x_2)$ and then restricted the resulting equation to $\boldsymbol{\gamma}(w)$. The intuitive reason for why this is true is that a system (stochastically) perturbed away from the slow manifold will return roughly along the flow lines of the ODE system when $\epsilon = 0$. But along these flow lines, by eq S6 we see that the proportion p remains constant.

To summarize our analysis to this point, we first specified a discrete state space stochastic process involving two types of competing individuals in eq S1. We then assumed that habitat size (and so population size) was sufficiently large that the discrete state space is approximately continuous, and obtained a diffusion approximation of the stochastic process (eq S3). We then applied the techniques of Parsons and Rogers [6] to derive a single-variable SDE approximating the dynamics of motion of eq S3 along the slow manifold by eliminating the dynamics on the fast timescale.

Speed measure and stationary distribution

As in the main text, let $\alpha(p) \equiv \mu(1-2p) - \epsilon c(p)p(1-p)$ and $\sigma^2(p) \equiv p(1-p)T(p)/[\Omega n(p)]$. Then $\alpha(p)$ and $\sigma^2(p)$ are the infinitesimal mean and variance, respectively, of a one-dimensional diffusion process [9, 10]; this is the diffusion process associated with the SDE S9. Define

$$\pi(p) \equiv \frac{C_0}{\sigma^2(p)} \exp\left(2 \int^p \frac{\alpha(z)}{\sigma^2(z)} dz\right) \tag{S10}$$

for some constant C_0. If C_0 can be chosen such that $\int_0^1 \pi(p)dp = 1$, then $\pi(p)$ is normalizable and so represents the stationary distribution of the diffusion process with infinitesimal mean $\alpha(p)$ and infinitesimal variance $\sigma^2(p)$ [1, 3, 10]. However, regardless of whether such a C_0 exists, $\pi(p)$ is the speed measure of the diffusion process [9]. The relevance of this is that the speed measure is proportional to the expected time a diffusion process initially at p takes to exit an interval $(\varepsilon - p, p + \varepsilon)$ for small ε [9]. Thus as $\pi(p)$

increases, the process will tend to spend more time at state p, and so we are more likely to observe the process in such a state. Hence as $\pi(p)$ increases for a given p, we will say state p is increasingly favoured.

There are three factors that influence eq S10:

1. selection, which is controlled by $\epsilon c(p)$, and biases the stochastic process against the social actor,
2. mutation rate, μ, which pushes $\pi(p)$ away from the boundaries ($p = 0$ and $p = 1$) towards $p = 1/2$,
3. and demographic stochasticity, which is controlled by the infinitesimal variance, $\sigma^2(p)$. If we inspect $\sigma^2(p)$, however, we see that it is the product of a symmetric term, $p(1-p)/\Omega$, and the ratio, $T(p)/n(p)$. Thus this ratio controls the effect of demographic stochasticity, and as we will see is key to understanding social trait evolution.

Evolution of cost-free social traits

To understand the role of demographic stochasticity, suppose that selection is turned off ($\epsilon = 0$). Then the only two factors present in eq S10 are mutation rate and the ratio $T(p)/n(p)$. First, consider how $T(p)/n(p)$ changes in p, that is,

$$\frac{d}{dp}\left[\frac{T(p)}{n(p)}\right] = \frac{T(p)}{n(p)}\left[\frac{dT/dp}{T(p)} - \frac{dn/dp}{n(p)}\right]. \tag{S11}$$

If the social trait is spite, $dn/dp < 0$, whereas if the social trait is altruism, $dn/dp > 0$. Since on the slow manifold, $T(p) = 2b(p) = 2m(p)$, if the social trait acts upon death rate, then $dT/dp = 2dm/dp > 0$ if the trait is spite, whereas if the trait is altruism, $dT/dp < 0$. Thus for social traits acting on the death rate, $T(p)/n(p)$ is monotonic in p, and so is either minimized by the social actor (if the trait is altruism) or the non-social actor (if the trait is spite). If instead the social trait acts upon birth rate, then since $dT/dp = 2db/dp$, if the trait is spite $dT/dp < 0$ and if the trait is altruism $dT/dp > 0$. Hence for both altruism and spite the ratio $T(p)/n(p)$ may be smaller for a population monomorphic for the social actor, or it may be smaller for a population monomorphic for the non-social actor. Moreover, it is also possible that $T(p)/n(p)$ is non-monotonic in p (and so minimized by a polymorphic population).

To understand the implications of the behaviour of $T(p)/n(p)$, first we will assume that $T(p)/n(p)$ is monotonic in p, and so is minimized by either type 1 or type 2, before considering what happens when $T(p)/n(p)$ is non-monotonic in p. There are three different regimes based upon mutation rate, and we consider each in turn.

1. **Low mutation rate**. When mutation rate is sufficiently low, then in the interval $p \in (0,1)$, $\pi(p) \approx 1/\sigma^2(p)$, and $\pi(p)$ is U-shaped. We are interested in whether $\pi(p)$ is increasing or decreasing in p. Taking the derivative gives

   $$\frac{d\pi}{dp} \approx \frac{d}{dp}\left[\frac{1}{\sigma^2(p)}\right] = -\frac{1}{\sigma^2(p)}\left(\frac{(1-2p)}{p(1-p)} + \frac{n(p)}{T(p)}\frac{d}{dp}\left[\frac{T(p)}{n(p)}\right]\right). \tag{S12}$$

   Since the first term, $(1-2p)/[p(1-p)]$, is symmetric in p, we can ignore it and instead focus upon how the ratio $T(p)/n(p)$ changes with respect to p. If the ratio is decreasing (resp. increasing) in p, then $d\pi/dp > 0$ (resp. $d\pi/dp < 0$), and the social actor is favoured (resp. disfavoured).

   An alternative way to arrive at this conclusion is to note that in the absence of selection (and mutations), the diffusion process is on its natural scale (i.e.,

$\alpha(p) = 0$), and so the fixation probability of either type is simply equal to its proportion in the population. Suppose there are k possible types of individuals, and let mutations be sufficiently rare such that between mutations the population returns to a monomorphic state. Then we can construct a Markov chain on the state space of possible strain types [11]. In particular, let μ_{ij} be the rate at which strain i mutates to strain j and N_i be the number of type i individuals in the population at the moment of the (rare) mutation. Then

$$M(i,j) = (\text{mutation rate}) \times (\text{fixation probability}) = \mu_{ij} N_i \times \frac{1}{N_i} = \mu_{ij} \qquad \text{(S13)}$$

is the rate at which the population transitions from a monomorphic strain i state to a monomorphic strain j state [11]. Hence in the absence of mutational biases, the Markov chain is equally likely to be in any particular state [11–13]. This implies that the process will spend equal time in any of the monomorphic states. But if so, then what type we are most likely to observe in the population will be dictated by the time the process takes to transition between states, that is, the expected time from the initial appearance of a mutation for the population to return to a monomorphic state. Using standard techniques [10], when there are two types in the population and they are selectively neutral, $\epsilon = 0$, then the expected time till absorption (time to reach either $p = 0$ or $p = 1$) from an initial state p_0 is

$$\bar{t}(p_0) = 2(1-p_0)\int_0^{p_0} \frac{\Omega n(p)}{(1-p)T(p)} dp + 2p_0 \int_{p_0}^{1} \frac{\Omega n(p)}{pT(p)} dp. \qquad \text{(S14)}$$

Since time spent in any monomorphic state is equal, the type that is most likely to be observed is the type which maximizes absorption time, that is, the type that maximizes the time spent transitioning between states.

To determine this, let ν control the effect of the social behaviour, and suppose that the effect of the social behaviour upon the ratio $T(p)/n(p)$ is small. Since the two types only differ due to the effects controlled by ν, we can write $T(p)/n(p) = T(\nu p)/n(\nu p)$, and so a Taylor expansion of the ratio $T(p)/n(p)$ with respect to ν gives

$$\frac{T(p)}{n(p)} = \frac{T}{n} + \nu p \left[\frac{\partial}{\partial[\nu p]} \frac{T(\nu p)}{n(\nu p)} \right]_{\nu=0} + \mathcal{O}(\nu^2). \qquad \text{(S15)}$$

If we then use this approximation for the ratio $T(p)/n(p)$ in the differential equation used to derive eq S14, we obtain the time till absorption as

$$\bar{t}(p_0) = -\frac{2\Omega}{T/n}\left(\ln([1-p_0]^{1-p_0} p_0^{p_0}) - \frac{\nu}{T/n}\frac{\partial T/n}{\partial[\nu p]} \ln([1-p_0]^{1-p_0}) \right) + \mathcal{O}(\nu^2), \qquad \text{(S16)}$$

where T/n no longer depends upon ν or p (or p_0). Notice from eq S16 that if the two types are identical in every respect, $\nu = 0$, then absorption time is symmetric in p about $p = 1/2$, and for any p, $\bar{t}(p)$ will decrease (resp. increase) as T/n becomes large (resp. small). Thus absorption time is maximized (resp. minimized) when we have minimized T/n (resp. maximized T/n). If instead $\nu > 0$, then time till absorption will no longer be symmetric about $p = 1/2$, and instead will depend upon how the social behaviour effects the ratio T/n. In particular, if we consider the difference $\bar{t}(p_0) - \bar{t}(1-p_0)$ on the interval $p_0 \in (0, 1/2)$, we see that this quantity will be negative, that is, the process will take longer to absorb from state $1 - p_0$ then p_0, provided the social trait minimizes the ratio T/n. In this

circumstance, it follows immediately that $\int_{1/2}^{1} \bar{t}(p_0)dp_0 / \int_0^1 \bar{t}(p_0)dp_0 > 1/2$. Hence if the social trait minimizes the ratio T/n, then absorption time will be biased in favour of the social actor and so the type minimizing T/n is favoured when mutations are sufficiently rare.

2. **Intermediate mutation rate**. Suppose mutation rate is non-negligible, but is not sufficiently high so as to admit a normalizable stationary distribution. Now the picture is more complex, and it is not clear how to determine which type is favoured. To see why, consider how $\pi(p)$ changes in p:

$$\begin{aligned}\frac{d\pi}{dp} &= 2\frac{\pi(p)}{\sigma^2(p)}\left(\alpha(p) - \frac{1}{2}\frac{d\sigma^2(p)}{dp}\right), \\ &= 2\frac{\pi(p)}{\sigma^2(p)}\Bigg(\underbrace{(1-2p)\left(\mu - \frac{T(p)}{2\Omega n(p)}\right)}_{(1)} - \underbrace{\frac{p(1-p)}{2\Omega}\frac{d}{dp}\left[\frac{T(p)}{n(p)}\right]}_{(2)}\Bigg).\end{aligned} \tag{S17}$$

From how the terms are grouped, we see that as $p \to 1/2$ term (1) disappears, whereas when $p \to 0$ or $p \to 1$, term (2) disappears (note it is also possible that there exists a p_0 such that $\mu = T(p_0)/[2\Omega n(p_0)]$, in which case term (1) also disappears as $p \to p_0$). Roughly speaking, this implies that term (1) is the stronger effect near the boundaries of the interval $p \in [0,1]$, whereas term (2) is the stronger effect in the interior. Term (1) represents the effect of mutations, μ, which pushes $\pi(p)$ towards $p = 1/2$, and genetic drift, $T(p)/[2\Omega n(p)]$, which pushes $\pi(p)$ towards the boundaries. These effects would be present even if the ratio $T(p)/n(p)$ were constant, that is, type 1 and 2 were mathematically interchangeable. Term (2), however, occurs due to how the magnitude of demographic stochasticity (or genetic drift) changes due to the consequences of the social trait, that is, how the ratio $T(p)/n(p)$ changes in p.

Consider the behaviour of $\pi(p)$ at the boundaries, and so focus upon term (1). When mutation rate satisfies $\mu < \frac{T(p)}{2\Omega n(p)}$ for all p, then the distribution tends to accumulate at both boundaries since genetic drift is stronger then mutations. As μ increases, however, because in general $\frac{T(0)}{2\Omega n(0)} \neq \frac{T(1)}{2\Omega n(1)}$, there will be a regime in which $\mu > \frac{T(p_1)}{2\Omega n(p_1)}$ but $\mu < \frac{T(p_2)}{2\Omega n(p_2)}$ where $\{p_1, p_2\} \in \{0,1\}$ with $p_1 \neq p_2$. Thus at the boundary which minimizes $T(p)/n(p)$ (p_1 boundary), mutations will be a stronger force then genetic drift, pushing the distribution towards the interior, whereas at the other boundary (p_2 boundary) the distribution will accumulate at the boundary in a state of quasi-fixation as the force of genetic drift outweighs mutations. This will tend to give rise to the sideways S-distribution as seen in Fig 1**e**,**f** in the main text.

This behaviour at the boundary prevents us from formulating a clear criteria about which type is 'favoured'. We can no longer focus exclusively upon the ratio $T(p)/n(p)$ as in the case of low mutation rate, but we also cannot use an integral measure to determine which type is stochastically favoured (i.e., does more of the mass of $\pi(p)$ occur for $p > 1/2$ or $p < 1/2$?), since $\pi(p)$ is not normalizable. However, because away from the boundaries the strongest effect upon the shape of $\pi(p)$ will be how the ratio $T(p)/n(p)$ changes in p (term (2) from eq S17), we may be inclined to argue that given sufficient 'segregating variation' exists, and so for polymorphic populations, the type minimizing the ratio $T(p)/n(p)$ will be favoured.

3. **High mutation rate**. Suppose mutation rate is sufficiently high such that the distribution is normalizable. Now we can compute $\int_{p_0}^{p_1} \pi(p)dp$, and so it makes

sense to use $\int_{1/2}^{1} \pi(p)dp$ to determine which type is favoured. In particular, if $\int_{1/2}^{1} \pi(p)dp > 1/2$, then the social actor is favoured (this assumes we have chosen C_0 such that $\int_0^1 \pi(p)dp = 1$). There are two possibilities here, either (a) mutations are of small effect, or (b) mutations are of large effect. We consider these cases in turn.

(a) Suppose mutations are of small effect, that is, type 1 only slightly differs from type 2 in terms of the social action. Let ν denote the social trait difference between types (ν may be positive or negative). This formulation allows for both types to be social actors: for example, if the social trait is altruism, then if $\nu > 0$ type 1 is more altruistic then type 2, while if $\nu < 0$, type 1 is less altruistic then type 2. To make it clear which quantities have a dependence on ν, we will explicitly include ν as an argument in the various functions, i.e., $T(p) = T(p, \nu)$. We will also write $\pi(p, \nu) = C_0(\nu)S(p, \nu)$, where

$$S(p, \nu) = \frac{1}{\sigma^2(p, \nu)} \exp\left(2 \int^p \frac{\alpha(z)}{\sigma^2(z, \nu)} dz\right),$$

and refer to $S(p, \nu)$ as the speed measure [9]. Hence, $C_0(\nu) = 1/\int_0^1 S(p, \nu)dp$, and $\frac{dC_0}{d\nu} = -C_0(\nu)^2 \int_0^1 \frac{\partial S}{\partial \nu} dp$. Note that when $\nu = 0$, both types are identical and so $T(p, 0)/n(p, 0) = T/n$ is constant with respect to p (since there are no costs), and so $\int_{1/2}^{1} \pi(p, 0)dp = 1/2$. Using this fact, a Taylor expansion of $\int_{1/2}^{1} \pi(p, \nu)dp$ gives

$$\begin{aligned}
\int_{1/2}^{1} \pi(p, \nu)dp &\approx \int_{1/2}^{1} \pi(p, 0)dp + \nu \int_{1/2}^{1} \left.\frac{\partial \pi}{\partial \nu}\right|_{\nu=0} dp + \mathcal{O}(\nu^2) \\
&= \frac{1}{2} + \nu \int_{1/2}^{1} \left[\frac{\partial S}{\partial \nu} C_0(\nu) + S(p, \nu)\frac{dC_0}{d\nu}\right]_{\nu=0} dp + \mathcal{O}(\nu^2) \\
&= \frac{1}{2} + \nu C_0(0) \int_{1/2}^{1} \left[\frac{\partial S}{\partial \nu} - \pi(p, \nu) \int_0^1 \frac{\partial S}{\partial \nu} dz\right]_{\nu=0} dp + \mathcal{O}(\nu^2) \\
&= \frac{1}{2} + \frac{\nu C_0(0)}{2} \left[\int_{1/2}^{1} \frac{\partial S}{\partial \nu} dp - \int_0^{1/2} \frac{\partial S}{\partial \nu} dp\right]_{\nu=0} + \mathcal{O}(\nu^2).
\end{aligned} \tag{S18}$$

The logic of eq S18 is clear: for example, if $\partial S/\partial \nu > 0$, then if the increase in the speed measure on the interval $(1/2, 1)$ exceeds the increase on the interval $(0, 1/2)$, we should expect the social actor to be favoured in the sense that $\int_{1/2}^{1} \pi(p, \nu)dp > 1/2$ since the process will tend to spend more time in the region $(1/2, 1)$.

To make further progress, we need to compute $\left.\frac{\partial S}{\partial \nu}\right|_{\nu=0}$. For ease of notation, let $R(p, \nu) \equiv T(p, \nu)/n(p, \nu)$ and $\omega \equiv \frac{2\Omega\mu}{R}$. Then

$$\frac{\partial S}{\partial \nu} = -S(p, \nu)\left[\frac{1}{R(p, z)} \frac{\partial R}{\partial \nu} + 2\Omega\mu \int^p \frac{1 - 2z}{z(1 - z)R(z, \nu)^2} \frac{\partial R}{\partial \nu} dz\right] \tag{S19}$$

Now what is $\partial R/\partial \nu$? Suppose we can write per-capita growth as $b(n) - d(n) + \nu\theta(n)np$, where $b(n)$ and $d(n)$ are the birth and death rates when $\nu = 0$, and $\theta(n)\nu$ is the (additional) social effect of type 1 individuals (which is multiplied by the density of type 1 individuals, np). The precise action of the social trait may be upon either the birth or death rate, while if $\theta(n)\nu < 0$, the trait

is spite whereas if $\theta(n)\nu > 0$, the trait is altruism. The function $\theta(n)$ controls any density-dependent effects of how the social action is distributed among members of the population.

Since on the slow timescale the process is in demographic equilibrium, per-capita growth is zero, and so at equilibrium

$$\nu p = \frac{b(n) - d(n)}{\theta(n)n}.$$

Let $G(n) \equiv [b(n) - d(n)]/[\theta(n)n]$, and assuming $G(n)$ is invertible, $n = g(\nu p) \equiv G^{-1}(\nu p)$. Thus we see that in order for $G(n)$ to be invertible, n must be a monotonic function of νp (which it is by our classification of the social traits). Since at equilibrium, $T(p,\nu)$ is either two times the per-capita birth rate or two times the per-capita death rate (e.g., if the social trait acts on birth rate, then the birth rate is $b(n)+\nu\theta(n)np$ and the death rate is $d(n)$ and thus $T(p,\nu) = 2(b(n) + \nu\theta(n)) = 2d(n))$, we can write R as a function of n, that is $R(n) = R(g(\nu p))$ (e.g., using our previous example, $R(n) = 2d(n)/n$). So all of the instances of ν in R are mediated through their presence in n. Then

$$\frac{\partial R}{\partial \nu} = \frac{\partial}{\partial \nu} R(g(\nu p)) = R'(n)g'(\nu p)p,$$

and when $\nu = 0$,

$$\left.\frac{\partial R}{\partial \nu}\right|_{\nu=0} = R'(g(0))g'(0)p = \delta p$$

where $\delta \equiv R'(g(0))g'(0)$ is constant with respect to p (and ν).

Using this information and the fact that

$$S(p,0) = \frac{\Omega}{R}\frac{1}{p(1-p)}\exp\left(\omega\int^p \frac{1-2z}{z(1-z)}dz\right) = \frac{\Omega}{R}\left(p[1-p]\right)^{\omega-1}$$

in eq S19 gives

$$\begin{aligned}\left.\frac{\partial S}{\partial \nu}\right|_{\nu=0} &= -\frac{S(p,0)}{R}\left(p + \omega\int^p \frac{1-2z}{z(1-z)}zdz\right)\delta \\ &= -\frac{S(p,0)}{R}\left(p + \omega\left[2p + \ln(1-p)\right]\right)\delta \\ &= -\frac{\Omega}{R^2}\left([1+2\omega]p + \omega\ln(1-p)\right)\left(p[1-p]\right)^{\omega-1}\delta. \qquad \text{(S20)}\end{aligned}$$

Now using eq S20 in eq S18 yields

$$\int_{1/2}^{1}\pi(p,\nu)dp \approx \frac{1}{2} - \frac{\nu C_0(0)}{2}\frac{\Omega\delta}{R^2}\left(\int_{1/2}^{1} f(p,\omega)dp - \int_0^{1/2} f(p,\omega)dp\right) + \mathcal{O}(\nu^2) \qquad \text{(S21)}$$

where

$$f(p,\omega) \equiv \left([1+2\omega]p + \omega\ln(1-p)\right)\left(p[1-p]\right)^{\omega-1}.$$

Since $\int_{1/2}^{1} f dp - \int_0^{1/2} f dp$ is a function of a single variable, ω, it can be plotted. From inspection of Fig A, this quantity is positive for $\omega > 0$ and goes to zero as $\omega \to \infty$. It follows that if $\delta\nu < 0$, type 1 is favoured, whereas if $\delta\nu > 0$, type 2 is favoured. But the sign of $\delta\nu$ has the same interpretation as before: whichever type minimizes the ratio T/n is favoured. Thus when mutations are of small effect, whichever type minimizes the ratio T/n is favoured.

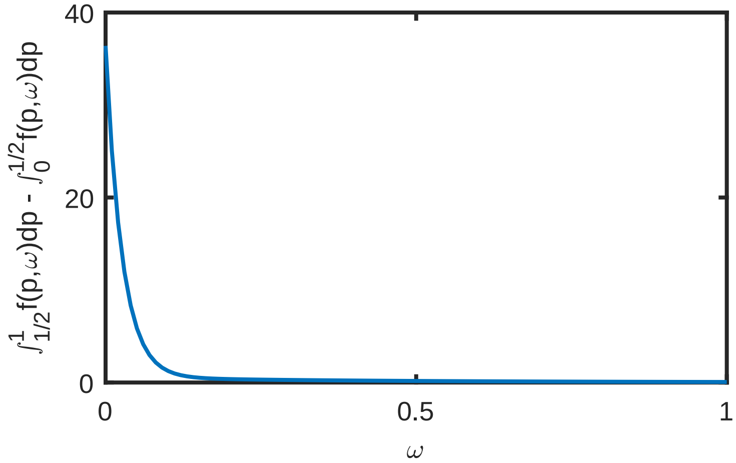

Fig A. As $\omega \to \infty$, $\int_{1/2}^{1} f dp - \int_{0}^{1/2} f dp \to 0$, but remains positive.

(b) If mutations are not of small effect, that is, $\nu \gg 0$, and mutation rate is also high, then mutations will exert a strong effect near the boundary pushing the weight of the distribution towards $p = 1/2$. In this regime, the ratio $T(p)/n(p)$ plays the strongest role. So provided $T(p)/n(p)$ is monotonic in p, then the type minimizing the ratio $T(p)/n(p)$ will tend to be favoured in the sense that the weight of the distribution will be displaced away from $1/2$ towards this boundary.

The preceding analysis assumed that the ratio $T(p)/n(p)$ was monotonic in p and so minimized at either $p = 0$ or $p = 1$. Suppose instead that $T(p)/n(p)$ is non-monotonic in p. Then for some $p \in (0, 1)$, say p^*, $\frac{d}{dp}\left[\frac{T(p)}{n(p)}\right] = 0$. From our analysis above, it is apparent where the issues are going to arise. For low mutation rate, when we are in a small neighbourhood of p^* consideration of eq S12 predicts that whichever type is more abundant will be favoured. Likewise, at intermediate mutation rates, now term (2) in eq S17 will be zero at p^* and so in a neighbourhood of p^* the dominant force shaping $\pi(p)$ will be term (1). But away from the boundary, whether term (1) favours or disfavours the social actor will depend both upon the value of p^* (is it greater or less than $1/2$?) and the magnitude of mutation rate relative to genetic drift at p^* (is μ greater or less than $T(p^*)/[2\Omega n(p^*)]$?). Finally, consider the case in which mutation rate is sufficiently high such that there is a normalizable distribution. If mutations are of small effect, that is, the two types are sufficiently similar in terms of the social trait, then implicit within the preceding analysis was that one of the types will minimize the ratio $T(p)/n(p)$ (and so $T(p)/n(p)$ is monotonic in p for sufficiently small ν). However, when mutations are of large effect, then consideration of eq S17 reveals again how the results become more complex and how the magnitude of mutation rate will alter the expected outcome.

Evolution of costly social traits

If we instead suppose that $\epsilon > 0$, then the social actor is selected against, and so can only be favoured if the influence of demographic stochasticity outweighs that of selection. This is similar to the observation from classical population genetics that in large populations, selection dominates, whereas in small populations, genetic drift does. Because of the inherent complexities owing to how the costs of the social traits are formulated (are

they density-dependent, or is $c(p)$ constant for all p?), as well as how the costs interact with mutations and demographic stochasticity, in the main text we simply focus upon numerical calculations to show that a stochastic reversal of selection is possible using the measure $\int_{1/2}^{1} \pi(p)dp$. If $\int_{1/2}^{1} \pi(p)dp > 1/2$, then despite the social actor being selected against, the population is most likely to be observed in a state in which the social actor is at greater frequency, and so we characterize this as a stochastic reversal of selection.

Examples

In this section we detail the calculations used to obtain the examples found in the main text.

1. $b(\mathbf{x}) \equiv \beta(1 - x_1 - x_2)$, $m(\mathbf{x}) \equiv d(1 + \nu x_1/[x_1 + x_2])$, and $c(\mathbf{x}) \equiv 0$ with $\beta > d(1 + |\nu|)$ and $\nu \in (-1, 1)$. Here the social action alters death rate: if $\nu < 0$ the trait is altruism, whereas if $\nu > 0$ the trait is spite. On the slow manifold, $w + \gamma_2(w) = (\beta - d(1 + \nu p))/\beta$, and so the ratio $T(p)/n(p)$ is

$$\frac{T(p)}{n(p)} = \frac{2\beta d(1 + \nu p)}{\beta - d(1 + \nu p)} \tag{S22}$$

   and thus

$$\frac{d}{dp}\left[\frac{T(p)}{n(p)}\right] = \frac{2\beta^2 d\nu}{(\beta - d(1 + \nu p))^2}, \tag{S23}$$

   which shares the same sign as ν. Thus if $\nu > 0$ then $T(p)/n(p)$ is increasing in p and so spite is disfavoured, whereas if $\nu < 0$ then $T(p)/n(p)$ is decreasing in p and so altruism is favoured. Simulations indicate these results extend to the n-type model (Fig 3).

2. $b(\mathbf{x}) \equiv \beta + \nu x_1$, $m(\mathbf{x}) \equiv d + \kappa_1(x_1 + x_2) + \kappa_2(x_1 + x_2)^2$ and $c(\mathbf{x}) \equiv 0$ with $\beta > d$ and $\nu > 0$. Here the social trait is altruism which alters birth rate. On the slow manifold, $\gamma_2(w) = (-2\kappa_2 w - \kappa_1 + \sqrt{4\kappa_2(\nu w + \beta - d) + \kappa_1^2})/(2\kappa_2)$, and so $h(p) = p(\nu p - \kappa_1 + \sqrt{(\nu p - \kappa_1)^2 + 4\kappa_2(\beta - d)})/(2\kappa_2)$. For this example, $T(p)/n(p)$ is non-monotonic in p (and ν) and so whether altruism is favoured or disfavoured depends upon the demographic parameters. In particular, the level of altruism minimizing $T(1)/n(1)$ is $\nu^* = (d\kappa_1 + \sqrt{\kappa_2 d(\beta - 2d)^2})/d$. Simulations predict this level of altruism also tends to be favoured in the n-type model (see Fig 3).

3. $b(\mathbf{x}) \equiv \beta(1 - \nu x_1/(x_1 + x_2 + a))$, $m(\mathbf{x}) \equiv d + \kappa_1(x_1 + x_2) + \kappa_2(x_1 + x_2)^2$ and $c(\mathbf{x}) \equiv 0$ with $\beta > d$, $a > 0$, and $\nu \in [0, 1]$. Here the social trait is spite which alters birth rate. In particular, with probability $\nu x_1/(x_1 + x_2 + a)$ a type 1 individual blocks another individual from reproducing. For this example, $\gamma_2(w)$ can be computed analytically, but the expression is unwieldy and so we do not show it here. Importantly, however, $T(p)/n(p)$ is a nonlinear function of ν, and the level of spite minimizing $T(1)/n(1)$ is

$$\nu^* = \left(a + \sqrt{\frac{d}{\kappa_2}}\right)\left(\beta - 2d - \kappa_1\sqrt{\frac{d}{\kappa_2}}\right)\sqrt{\frac{\kappa_2}{\beta^2 d}}.$$

   Simulations predict this level of spite also tends to be favoured in the n-type model (Fig 3).

4. $b(\mathbf{x}) \equiv r + \nu x_1$, $m(\mathbf{x}) \equiv \kappa(x_1 + x_2)$, and $c(\mathbf{x}) \equiv r$, with $r, \nu, \kappa > 0$ and $\kappa > \nu$. Here the social trait is altruism which increases birth rate. On the slow manifold,

$\gamma_2(w) = (r - [\kappa - \nu]w)/\kappa$, and so $h(p) = rp/(\kappa - \nu p)$. Using this information yields the stationary distribution

$$\pi(p) \propto p^{\mu\Omega/\kappa - 1}(1-p)^{\mu\Omega/\kappa - 1} e^{-\epsilon r \Omega p/\kappa}, \tag{S24}$$

where proportionality is up to a positive constant. In the absence of costs ($\epsilon = 0$), eq S24 is symmetric about $p = 1/2$, and so neither type is stochastically favoured. The reason for this result is that $T(p)/n(p) = 2\kappa$ which is constant for all p.

5. $b(\mathbf{x}) \equiv \beta + \nu x_1$, $m(\mathbf{x}) \equiv d + \kappa(x_1 + x_2)$, and $c(\mathbf{x}) \equiv r$, with $\beta > d > 0$, $\kappa > \nu > 0$ and $r = \beta - d$, and so the social trait is altruism increasing the birth rate. The per-capita growth rate in this model is the same as in example 4 and so $\gamma_2(w)$ and $h(p)$ are the same. However now the stationary distribution is

$$\pi(p) \propto p^{\frac{\mu\Omega r}{\beta\kappa} - 1}(1-p)^{\frac{\mu\Omega r}{\beta\kappa - d\nu} - 1}(\beta\kappa - d\nu p)^{\frac{r^2\Omega\epsilon}{d\nu} - \frac{\mu\Omega r}{\beta\kappa} - \frac{\mu\Omega r}{\beta\kappa - d\nu} - 1}.$$

   Here, in the absence of costs, the distribution is asymmetric and favours type 1 (the altruist). This can be seen by noticing that $T(p)/n(p) = 2(\beta\kappa - d\nu p)/r$, which is decreasing in p.

Interestingly, example 4 is the non-spatial model of Constable, Rogers, McKane & Tarnita [8] (CRMT), differing only in that we have explicitly included mutations (our notation also slightly differs). CRMT concluded that stochasticity induced an advantage for the altruist whereas our analysis shows altruism is stochastically neutral if cost-free and disfavoured otherwise. There are two reasons for this discrepancy.

1. Density versus frequency. Rather than dealing with the SDE for proportions directly (eq S9), CRMT focused upon interpreting the infinitesimal mean of the density SDE, dx_1 (this can be obtained from (S9) by application of Ito's formula [1]). However, evolution is a change in frequency, not density, and a change in density is not equivalent to a change in frequency. This is clear from eq S9 where in the absence of mutations and selection, the infinitesimal mean is zero while $\sigma^2(p) = \sqrt{\frac{2\kappa}{\Omega}p(1-p)}$ so the stochastic process is mathematically equivalent to pure genetic drift in a population of constant size [14].

2. Inclusion of mutations. At selective neutrality, which is the scenario most conducive to the evolution of altruism, the fixation probability of a particular type is equal to its proportion in the population. Thus the invasion probability of a type j mutant into a monomorphic type i population of size N_i is simply $1/N_i$. As the altruist can grow to a larger population size then the non-altruist, pairwise comparison of invasion probabilities predicts the altruist is favoured [8, 15, 16]. However, if we assume type i mutates to type j at a per-capita rate μ_{ij}, then as shown in eq S13 the transition rate from an all-type i state to an all-type j state is μ_{ij}. So in the absence of mutational biases, the Markov chain is equally likely to be in any state, a standard result for neutral evolution in sequential-fixation models [11–13]. Hence mutations erase any numerical advantage of the altruists. However, this does not take into account that in our model the expected time till fixation varies based upon population composition. Consideration of expected time till fixation (or absorption time) reveals the importance of the ratio $T(p)/n(p)$ (see eq S16).

Simulations

To support our analytic predictions, we use two types of simulations: Gillespie's algorithm [17] and the Euler-Maruyama (EM) method [3]. In particular, for plots involving the

stationary distribution of the two-type model, we have used Gillespie's algorithm (Fig 1, Fig 2 and Fig 4) to simulate the full stochastic process specified by eq S1. When we consider more then two types (as in Fig 3) we simulate the system of stochastic differential equations which approximates eq S1 using the EM method [3], which we detail briefly here. For the EM method, we assume that a type i individual mutates to a type j individual at rate μ. Thus the total rate at which a type i individual mutates to a *different* type is $\mu(n-1)$. Let η be a $n \times n$ matrix of standard normal random variables, and let $\Delta\tau$ be the step-size. Then using EM, the change in variable x_i over the time increment $\Delta\tau$, i.e., $\Delta x_i \equiv x_i(\tau + \Delta\tau) - x_i(\tau)$, is given by

$$\begin{aligned}\Delta x_i = &\big((b_i(\mathbf{x}) - m_i(\mathbf{x}) - \mu n)x_i + \sum_j \mu x_j\big)\Delta\tau \\ &+ \sqrt{\frac{(b_i(\mathbf{x}) + m_i(\mathbf{x}))x_i\Delta\tau}{\Omega}}\eta_{ii} + \sum_j \left(\sqrt{\frac{\mu x_j \Delta\tau}{\Omega}}\eta_{ij} - \sqrt{\frac{\mu x_i \Delta\tau}{\Omega}}\eta_{ji}\right). \quad \text{(S25)}\end{aligned}$$

For all the n-type simulations used in this paper, $\Delta\tau = 0.01$, $\Omega = 10^4$, and $\mu = 10^{-6}$, and the initial conditions were chosen to be $x_i(0) = 0.05$ for all i; this was then simulated until $\tau = 2 \times 10^6$ for 10^4 individual simulations (we checked that the distribution had settled down by $\tau = 2 \times 10^6$). Then if $x_i^{(j)}(\tilde{\tau})$ is the density of type i in sample run j at time $\tilde{\tau}$, then the probability of type i plotted in Fig 3 (black circles) is

$$\mathrm{Prob}(x_i) = \sum_j \frac{x_i^{(j)}(\tilde{\tau})}{\sum_k x_k^{(j)}(\tilde{\tau})} \Bigg/ \left[\sum_\ell \sum_j \frac{x_\ell^{(j)}(\tilde{\tau})}{\sum_k x_k^{(j)}(\tilde{\tau})}\right]. \quad \text{(S26)}$$

The primary reason for using EM method rather than Gillespie's algorithm when the number of types is greater then 2 is that the computational costs of Gillespie's algorithm rapidly become prohibitive. This is because when we construct the stationary distribution in the 2-type case, in order to have the distribution normalizable, mutations must be artificially high. When mutations are high, the population composition can change more rapidly, and so simulations reach the stationary distribution more rapidly. When we extend the system to include more than 2-types, mutations have a homogenizing effect, and so we lower the mutation rate. However, this means that the population composition changes less quickly, and so more time must elapse to obtain the stationary distribution.

References

1. Gardiner CW. Handbook of Stochastic Methods. Berlin, Germany: Springer: Complexity; 2009.

2. van Kampen NG. Stochastic Processes in Physics and Chemistry. New York, NY: North-Holland; 1981.

3. Allen LJS. An Introduction to Stochastic Processes with Applications to Biology. Boca Raton, FL: CRC Press; 2011.

4. Carr J. Applications of Centre Manifold Theory. New York, NY: Springer-Verlag; 1981.

5. Berglund N, Gentz B. Noise-induced Phenomena in Slow-Fast Dynamical Systems. USA: Springer; 2006.

6. Parsons TL, Rogers T. Dimension reduction for stochastic dynamical systems forced onto a manifold by large drift: a constructive approach with examples from theoretical biology. J Phys A: Mathematical and Theoretical. 2017;50.

7. Constable GWA, McKane AJ, Rogers T. Stochastic dynamics on slow manifolds. J Phys A: Math Theor. 2013;46.

8. Constable GWA, Rogers T, McKane AJ, Tarnita CE. Demographic noise can reverse the direction of deterministic selection. Proc Nat Acad Sci. 2016;.

9. Karlin S, Taylor HM. A Second Course in Stochastic Processes. New York, NY: Academic Press; 1981.

10. Ewens WJ. Mathematical Population Genetics I: Theoretical Introduction. New York, NY: Springer: Interdisciplinary Applied Mathematics; 2004.

11. McCandlish DM, Stoltzfus A. Modeling evolution using the probability of fixation: history and implications. Quarterly Review of Biology. 2014;89:225–252.

12. Kimura M. Evolutionary rate at the molecular level. Nature. 1968;217:624–626.

13. King JL, Jukes TH. Non-Darwinian evolution. Science. 1969;164:788–798.

14. Kimura M. Solution of a process of random genetic drift with a continuous model. Proc Natl Acad Sci. 1955;41:144–150.

15. Houchmandzadeh B, Vallade M. Selection for altruism through random drift in variable size populations. BMC Evol Biol. 2012;12.

16. Houchmandzadeh B. Fluctuation driven fixation of cooperative behavior. Biosystems. 2015;127:60–66.

17. Gillespie DT. Exact stochastic simulation of coupled chemical reactions. J Phys Chem. 1977;81:2340–2361.
